# Diet-Induced Obesity Alters the Circadian Expression of Clock Genes in Mouse Gustatory Papillae

**DOI:** 10.3389/fphys.2020.00726

**Published:** 2020-06-30

**Authors:** Arnaud Bernard, Aurélie Dastugue, Guillaume Maquart, Stéphane Delhaye, Hélène Duez, Philippe Besnard

**Affiliations:** ^1^UMR Lipide/Nutrition/Cancer, 1231 Inserm/University Bourgogne Franche-Comté, Dijon, France; ^2^Univ. Lille, Inserm, CHU Lille, Institut Pasteur de Lille, U1011-EGID, Lille, France

**Keywords:** diet-induced obesity, circadian rhythm, gustatory papillae, orosensory sensitivity to lipids, taste sensitivity

## Abstract

Diet-induced obesity (DIO) is associated with a defect of the orosensory detection of dietary lipids in rodents. This dysfunction is not anecdotic since it might worsen the negative effects of obesity by promoting the overconsumption of energy-dense foods. Previous studies have highlighted a progressive devaluation of reward value of lipid stimuli due to a desensitization of dopaminergic brain areas in DIO mice. Paradoxically, the putative deleterious impact of obesity on peripheral fat detection by the gustatory papillae remains poorly documented. Using a whole transcriptomic investigation of the circumvallate papillae (CVP), an analysis of CVP genes involved in fat taste transduction and signaling along the day, and two bottle choice tests, we have found that (i) CVP, known to house the most taste buds in the oral cavity, displays a genic circadian rhythm, (ii) DIO reduces the oscillation of key genes involved both in the circadian clock and lipid detection/signaling, and (iii) the gene invalidation of the clock gene Rev-Erbα does not significantly affect fat preference despite an oily solution intake slightly lower than littermate controls. Taken together these data bring the first demonstration that the gustatory function is under control of a peripheral clock in mammals, as already reported in fly and suggest that a disturbance of this rhythmicity might contribute to the lower fatty taste acuity found in obese mice.

## Introduction

The dietary habits deeply impact health and thereby quality of life. This behavior is clearly multifactorial depending on environmental factors (cultural values, social influences, conveniences, price) and physiological determinants (genetic traits, metabolic needs, sensory appeal, expected pleasure). Among the sensory qualities, gustation appears to be a critical factor guiding the food choices. Therefore, a disturbance of the taste sensitivity could affect the food preference. Consistently, diet-induced obesity (DIO) renders rats and mice unable to detect properly low concentrations of oily solutions (Shin et al., 2011a; Chevrot et al., 2013). How obesity affects fatty taste sensitivity is not fully elucidated.

At the central level, the forebrain gustatory relays have connections with nucleus accumbens (NAc) known to be a dopaminergic area implicated in the reward pathway (Norgren et al., 2006). In rats, sham licking of corn oil produces dopamine release by the NAc suggesting that oral lipid stimulation leads to activation of the rewarding pathway (Liang et al., 2006). By contrast, dopamine response to oral lipid stimuli is blunted in DIO rats (Johnson and Kenny, 2010). The physiological consequence of this obesity-induced NAc desensitization might be a progressive devaluation of reward value of the oral lipid stimuli, as found with abuse drugs (Volkow et al., 2013). This reward deficiency might explain the susceptibility to overeating energy-dense foods observed in DIO animals (Shin and Berthoud, 2011), probably to gain the desired hedonic response (Johnson and Kenny, 2010).

In periphery, three types of gustatory papillae (i.e., fungiform, foliate and circumvallate – CVP), mainly located on the dorsal tongue, are responsible for the generation and the transfer of taste signals to the brain. Paradoxically, their respective implication in the reduction of fatty taste acuity in the context of obesity remains poorly documented. *In vitro*, the intracellular ionized calcium responses to linoleic acid stimuli and the subsequent release of neurotransmitters are lower in freshly isolated taste bud cells from DIO mice than from lean controls (Ozdener et al., 2014). However, origin of this functional alteration remains unclear and its impact on the food choice unknown. A pro-inflammatory gene profile was found in the CVP from DIO mice (Bernard et al., 2019). Interestingly, inflammation induced by the chronic intake of high-fat diet (HFD) is associated with a reduction of the fungiform density in obese mice (Kaufman et al., 2018). However, whether this change affects the fatty taste sensitivity remains to be determined. Moreover, the induction of a chronic low-grade inflammation similar to that seen in obese mice fails to reproduce in lean mice the impairment of preference for oily solution (Bernard et al., 2019). Therefore, it is likely that the obesity-mediated defect of the orosensory fat detection results from a more complex systemic influence than previously expected. The purpose of the present study was to explore this issue further by using a whole transcriptomic analysis of CVP from DIO and control mice and a targeted gene expression analysis. We found that CVPs genes involved in the lipid signaling exhibited a diurnal rhythm, this regulation being altered in DIO mice.

## Materials and Methods

### Animals

This study was carried out in the strict accordance with European guidelines for the care and use of laboratory animals and protocol approved by the French National Animal Ethic Committee (CNEA n°105). Six-weeks-old C57Bl/6 male mice were purchased from Charles River Laboratories (France). Animals were individually housed in a controlled environment (constant temperature and humidity, dark period from 7 p.m. to 7 a.m.) and had free access to tap water and chow. Experiments took place after a 1-week acclimatization period. *Rev-Erb*α^–/–^ mice and their wild-type littermates were obtained from B. Vennström, and backcrossed >8 generations with SV129 mice. Three complementary studies were performed: (i) a transcriptomic analysis of the circumvallate papillae (CVP), (ii) exploration of genes rhythmicity in CVP, and (iii) preference tests in Rev-Erbα-null mice. Nutritional obesity was induced by feeding *ad libitum* a saturated HFD (33% w/w palm oil) for 17 weeks (Bernard et al., 2019). Fat mass was determined by molecular resonance imaging (EchoMRI – Echo Medical Systems, Houston, TX, United States).

### Two-Bottle Choice Tests

Experiments were performed for 12 h at the beginning of the dark phase in individually housed mice. Animals were food restricted during the duration of the experiment. Mice were subjected to a choice between a control solution (2% xanthan gum in water w/v) and an experimental one (2% rapeseed oil w/v, suspended by agitation in the control solution). At the end of the test, fluid intake was measured for each bottle and the preference (i.e., ratio between experimental solution consumption and total intake) was calculated.

### Tissue Collection

The single CVP found in the posterior part of dorsal tongue of mice was isolated according to the procedure described elsewhere (Laugerette et al., 2005). Briefly, after separation of the lingual epithelium from subjacent connective tissue by enzymatic dissociation (elastase and dispase mixture, 2 mg/ml each in Tyrode buffer, pH 7.4), the CVP was thoroughly dissected under a binocular microscope then stored at −80°C until assays.

### Transcriptomic Analysis

Transcriptomic analysis was performed by the Get-TRIX platform (INRA, Toulouse, France) using Agilent Sureprint G3 Mouse microarrays (8 × 60K, design 028005). For each sample, Cyanine-3 (Cy3) labeled RNA was prepared from 25 ng of total RNA using the One-Color Quick Amp Labeling kit (Agilent), followed by Agencourt RNAClean XP (Agencourt Bioscience Corporation, Beverly, MA, United States). Cy3-lebeled RNA (600 ng) was hybridized on a microarray slide. After washing, the slides were scanned on Agilent G2505C Microarray Scanner using Agilent Scan control A.8.5.1 software and the fluorescence signal was extracted using Agilent Feature (extraction software v10.10.1.1 with default parameters). One sample from lean controls didn’t pass the quality check and was excluded from the analysis. Microarray data and experimental details are available in the Gene Expression Omnibus (NCBI-GEO) database (accession GSE111719).

### Real-Time Polymerase-Chain Reaction

Total RNA of CVP were extracted using a total RNA purification kit (Norgen Biotek, Canada). Briefly, the nitrogen-frozen CVP were homogenized in the lysing buffer, and after a selection on columns, RNA previously treated with a DNase (RNase-free DNase I kit, Norgen Biotek, Canada) were assayed using a nanodrop spectrometer (Thermo Fischer Scientific). RT-PCR were performed using the following primers (Life Technologies, Thermo-Fisher, France): Arntl/Bmal1, Mm00500223m1; CLOCK, Mm00455950m1; Per2, Mm00478099m1; Cry2, Mm01331539m1; NR1D1/RevErbα, Mm00520708m1; NR1D2/RevErbβ, Mm01310356g1; CD36, Mm00432403m1; GPR120/FFAR4, Mm00725193m1; PLCβ2, Mm01338057m1; Tas1R3, Mm00473459g1.

### Statistical Analysis

The statistical analysis was performed using R 3.4.4. with an alpha level of 0.05. According to the little size of samples, non-parametric tests were used. Mean comparisons were realized with Mann–Whitney and Wilcoxon tests.

## Results

### DIO Decreases the Preference for Lipids and Affects the Expression of Clock Genes in the CVP

**FIGURE 1:**
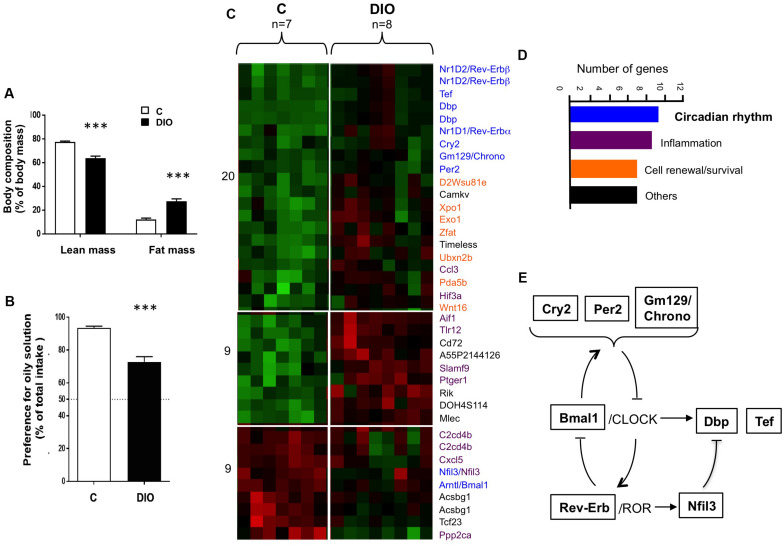
DIO affects the preference for lipids and the expression of genes involved in the diurnal rhythm in the circumvallate gustatory papillae (CVP). **(A)** Comparison of lean and fat mass in lean controls (C) and diet-induced obese (DIO) mice. **(B)** Preference for oil solution (2% rapeseed oil, v/v) during two-bottle preference test. **(C)** Heat map generated by the transcriptomic analysis of CVP. Increasing brightness indicates the relative fold-change rise (red) or drop (green) of gene expression. **(D)** Functional role and number of genes differentially expressed. **(E)** Simplified inter-relations of circadian genes identified. Means ± SEM. ^∗∗∗^*p* < 0.001.

High-fat diet fed mice displayed a 3-fold rise in body fat mass as compared to age-matched controls fed the standard laboratory chow (Figure 1A). Consistent with our previously published data (Chevrot et al., 2013), DIO mice showed a lower preference for oily solution (2% rapeseed oil, wt/wt) than lean controls (C), when they were subjected to a long-term (12 h) two-bottle preference test (Figure 1B). To determine whether this change could be partly explained by a functional impairment of the oral fat detection, a transcriptomic analysis of CVP freshly isolated from C and DIO mice was undertaken. CVP was chosen because it houses most the taste buds found upon dorsal tongue. Among the genes differentially expressed (Figure 1C), seven encode proteins implicated in cell renewal and survival, eight for proteins involved in the inflammatory process (Bernard et al., 2019), and nine for clock or clock-controlled genes (Figure 1D). Indeed, Bmal1, Cry2, Per2, Rev-Erbα, Rev-Erbβ, and Nfil3 are keys players of the core-clock network, whereas Dbp, Tef and Gm129 (also termed Chrono) are clock-controlled output genes (Figure 1E). This last observation suggests that the circadian clock machinery might modulate the physiological activities of gustatory papillae in the mouse, DIO altering this regulation.

### DIO Reduces the Rhythmic Pattern of Clock Genes in the CVP

**FIGURE 2:**
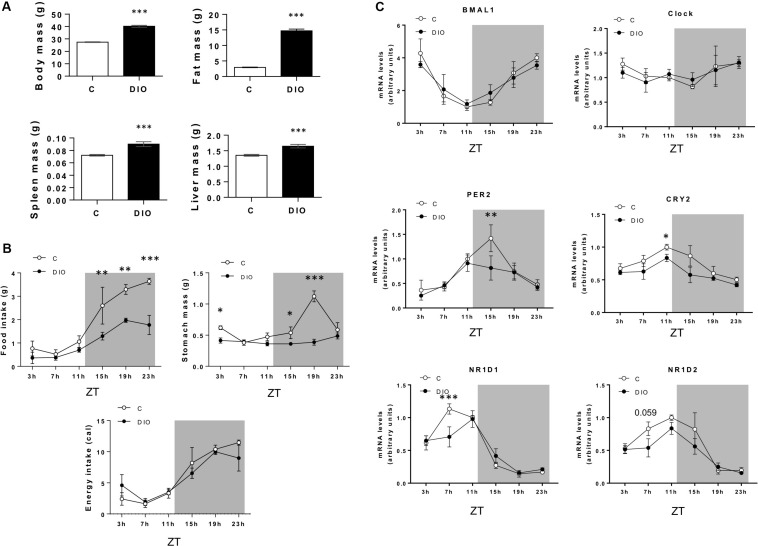
DIO reduces the rhythmic pattern of clock genes in the circumvallate papillae (CVP). **(A)** Comparison of body composition of lean controls (C) and diet-induced obese (DIO) mice. **(B)** Daily feeding pattern. **(C)** Diurnal expression of clock genes in the CVP. Independent groups of five mice by slot time. ZT, zeitbeger. Means ± SEM. *p < 0.05; **p < 0.01; ***p < 0.001.

Next, expression of genes encoding proteins involved in the molecular core clock machinery was studied every 4 h throughout a day/night cycle in CVP from controls and DIO mice (*n* = 5/time point). Analysis of the body composition clearly distinguished DIO mice from lean C (Figure 2A). Despite the protocol used (independent groups of five mice by slot time), significant differences in the feeding pattern were found. While the food consumption and the stomach weight were similar during the light period in the two groups, they were significantly lower in DIO mice during the dark phase. Despite this difference in total gram of intake, the DIO and control mice have eaten the same number of calories (Figure 2B). As Expected, expression of the core-clock genes displayed circadian oscillations in CVP, the expression of Bmal1 being lower level being reached at the end of the light phase (i.e., ZT11), while the expression of Clock remained unchanged across the 24-h cycle (Figure 2C), similarly to what was found in hypothalamus (Kohsaka et al., 2007). Per2 and Cry2, known to be regulated by the Bmal1/Clock complex (Figure 1E), showed an opposite pattern and Nr1d1 and Nr1d2 mRNA, encoding the transcription factors Rev-Erbα and Rev-Erbβ, respectively, were higher toward the end of the light phase and progressively dropped during the dark period (Figure 2C). Interestingly, the amplitude of oscillations of Per, Cry, and Nr1d genes tended to be reduced in obese mice, suggesting that a chronic consumption of a saturated HFD alters the molecular circadian rhythms of gustatory papillae in this species (Figure 2C).

### DIO Disturbs the Diurnal Rhythm of Lipid Sensing System in the CVP

**FIGURE 3:**
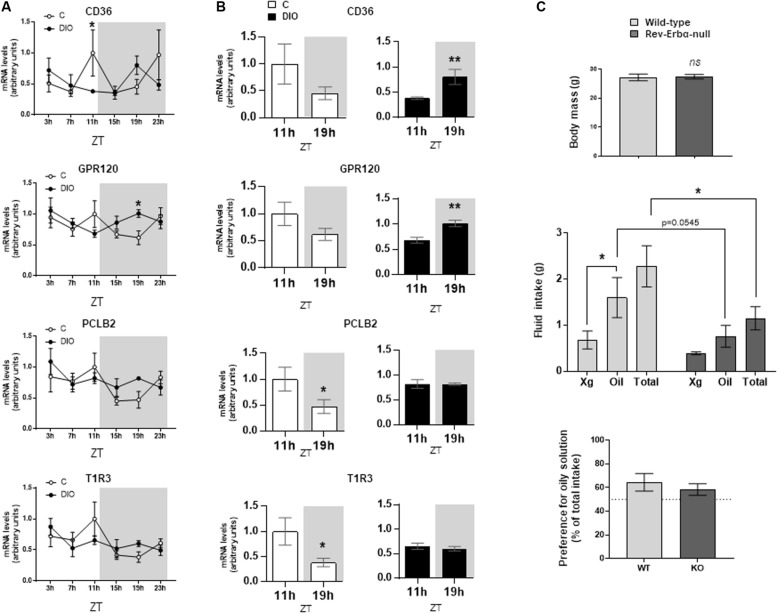
DIO disturbs the diurnal rhythm of lipid sensing system in the circumvallate papillae (CVP), but fat preference is not affected in the Rev-Erbα-null mice. **(A)** Diurnal expression of key genes for the transduction of fat and sweet signals in the CVP from lean controls (C) and diet-induced obese (DIO) mice. **(B)** mRNA levels at the end of the light period (ZT11) and the middle of the night (ZT19). **(C)** Two-bottle preference test in wild-type and Rev-Erbα-null mice. Xg, control solution (0.3% xanthan gum in water). Oil, experimental solution (2% rapeseed oil in the control solution). ZT, zeitbeger. Means ± SEM. ^∗^*p* < 0.05; ^∗∗^*p* < 0.01, ns, non significant.

The orosensory detection of dietary lipids is elicited by the binding of long-chain fatty acids to specific lipid receptors CD36 and GPR120 (FFAR4) found in the apical side of the taste bud cells (Laugerette et al., 2005; Cartoni et al., 2010). This lipid-receptor interaction triggers a signaling cascade initiated by the activation of PLCβ2 [for a review, see Besnard et al. (2016)]. To explore whether the obesity-induced reprogramming of the CVP clock is associated with a change in the expression of genes involved in lipid detection and signaling, CD36, GPR120, and PLCβ2 mRNA levels were analyzed along the day/night cycle in CVP from C and DIO mice. Expression profiles of these genes showed a rhythmicity in C mice with a peak occurring at ZT11 followed by a decrease reaching a minimum at ZT19 (Figures 3A,B), when satiety is gradually developing. This expression pattern was also found for the T1R3 gene involved in the sweet taste detection (Figure 3B). By contrast, these diurnal mRNA oscillations were blunted in DIO mice. Comparison of mRNA levels between ZT11 and ZT19 showed either no change for PLCβ2, T1R3 or even an increase at ZT19 for CD36, GPR120 (Figure 3B).

### Preference for Lipids Is Unchanged in Rev-Erbα-Null Mice

Targeted brain mutation of Rev-Erbα prevents the food-anticipatory behavior, alters lipid metabolism and leads to obesity (Delezie et al., 2016). Therefore, Rev-Erbα appears to be not only an integrator of circadian rhythm and metabolism (Duez and Staels, 2009), but also a significant player of the eating behavior regulatory network. To explore whether an alteration of the CVP circadian rhythmicity might alter the orosensory perception of lipids, two-bottle choice tests were performed in Rev-Erbα–muted mice. A lower consumption of oily solution was found when Rev-Erbα was lacking (Figure 3C). It is unlikely that this orosensory change was elicited by a difference in fat stores, wild-type and Rev-Erbα muted mice displaying a similar body weight (Figure 3C). However, this behavioral change did not significantly affect the lipid preference, null-mice also drinking less the control solution (Figure 3C).

## Discussion

Orosensory sensitivity to lipid stimuli is compromised in DIO rodents (Shin et al., 2011a; Chevrot et al., 2013; Bernard et al., 2019). This peripheral sensory distortion, associated with a central neuro-gustatory vulnerability, usually renders high fat foods more attractive (Shin et al., 2011b), probably to reach an hedonic fulfillment (Besnard, 2016). Although this food choice worsens obesity, underlying mechanisms remain poorly understood.

In the present study, we report the existence of a rhythmic expression of genes involved in both the circadian rhythm and the lipid sensing in the mouse CVP. To our knowledge, it is the first demonstration that the gustatory function is under control of a peripheral clock in mammals, as already reported in fly. In the *Drosophila melanogaster*, the variation of circadian clock gene expression in the gustatory receptor neurons on the proboscis (i.e., the main gustatory organ in this species) affects the feeding behavior by modulating the taste sensitivity along a day (Chatterjee and Hardin, 2010). Clock genes increase taste sensitivity in the morning, facilitating food-detection ability and, thereby, the feeding, while they decrease it at night, during the fasting period (Chatterjee and Hardin, 2010). This regulatory loop likely represents an advantage inherited from evolution, which might come very useful in a context of food scarcity. Consistent with this paradigm, our data show that the expression levels of genes involved in the transduction of fat and sweet signals in taste bud cells (CD36, GPR120, PLCβ2, and T1R3) are higher in the CVP from lean controls at ZT11 (i.e., high detection sensitivity when food intake is low and intermittent) than at ZT19 (i.e., low detection sensitivity at a time where satiety is gradually reached). For CD36, the drop of mRNA during the dark period seems to be consistent with the reduction of CD36 protein level observed in mouse CVP following food intake (Martin et al., 2011). Collectively these data strongly suggest that the taste sensitivity changes along the day in the mouse as in the fly. By extension, such a functional oscillations of gustatory papillae might also explain why acuity to sweet and salty varies in diurnal manner in human, the highest sensitivity occurring in the morning after a night fasting (Fujimura et al., 1990; Nakamura et al., 2008).

The second important finding is that the nutritional obesity interferes with the CVP rhythmicity by decreasing the amplitude of clock gene oscillations. Such a phenomenon has already been observed in other tissues in this species. For example, variation of Bmal-1 expression was reduced in adipose tissue and liver in both light and dark periods in obese mice (Kohsaka et al., 2007). In the CVP, the down-regulation of the lipid sensing genes (CD36, GPR120) occurring in the middle of the dark period in lean controls was not found in DIO mice suggesting that their taste sensitivity remains high despite HFD consumption. Disruption of this regulatory loop might play a role in the preferential consumption of energy-dense foods observed in DIO mice (Shin et al., 2011b). This assumption raises the question of the underlying mechanism. A body of evidence from literature identifies the incretin GLP-1 as a plausible candidate. First, the secretion of GLP-1 displays a rhythmic pattern with peak occurring at the beginning of active/feeding period in the mouse (Biancolin et al., 2020). Second, an obesogenic diet rich in saturated fatty acids leads to the disruption of GLP-1 rhythm (Gil-Lozano et al., 2016). Third, the food-induced decrease of CD36 protein in CVP is lacking in GLP-1-null mice (Martin et al., 2011). As GLP-1 contributes to the regulation of the sweet taste sensitivity (Martin et al., 2009), the deregulation of its rhythm by DIO might explain the loss of sweet taste sensitivity observed in obese subjects (Sanematsu et al., 2018).

To explore further the link between the molecular clock system and the fatty taste, Rev-Erbα-null mice were subjected to two-bottle preference tests. Indeed, Rev-Erbα is required to integrate feeding cues controlling the eating behavior including taste-guided food choice (Hariri and Thibault, 2011; Delezie et al., 2016). Moreover, Rev-Erbα gene expression levels in mouse CVP diminishes during the dark phase, similar to lipid sensing genes (Figures 2C, 3A). Rev-Erbα-null mice displayed a lower consumption of oily solution than wild-type littermate controls (*p* = 0.054), suggesting that the absence of Rev-Erbα reduced the lipid appetite. However, this change was insufficient to decrease significantly the preference for oily solution, the consumption of the control solution being also reduced in Rev-Erbα-null mice. This inconclusive data might be due to the presence of Rev-Erbβ which is known to share redundant function with Rev-Erbα (Liu et al., 2008). Moreover, the preference for the 2% oily solution is unusually low in KO mice and their controls (around 60%, Figure 3D) as compared to what is commonly found (Figure 1B). The origin of this discrepancy is not yet known, but might be related to the different genetic backgrounds, these mice having been backcrossed with SV129 mice while the others mice used in this study were C57Bl6 mice. Indeed, 129X1/SvJ derived from SV129 strain display specific taste phenotypes as compared to C57Bl6 (Tordoff, 2007). Further experiments are required to fully explore this question.

In conclusion, these data bring the first demonstration that circumvallate gustatory papillae exhibits a daily rhythm in the mouse and that nutritional obesity disturbs the oscillation pattern of genes involved in lipid sensing. The fact that these findings were obtained despite a limited number of animals (only five mice/time for ethic reason) argues in favor of their physiological relevance. Better understanding of regulatory mechanisms responsible for the functional plasticity of gustatory papillae and, thereby, their roles on taste-guided food choices might lead to new pharmacological strategies facilitating compliance with healthy dietary recommendations associated with anti-obesity treatments.

## Data Availability Statement

The datasets presented in this study can be found in online repositories. The names of the repository/repositories accession number(s) can be found below: https://www.ncbi.nlm.nih.gov/geo/query/acc.cgi?acc=GSE111719.

## Ethics Statement

The animal study was reviewed and approved by the French National Animal Ethic Committee (CNEA n°105).

## Author Contributions

AB and PB contributed to conception and design of the study. AB, AD, GM, SD, HD, and PB performed the experiments. AB and AD performed the statistical analysis. PB wrote the first draft of the manuscript. HD wrote sections of the manuscript. All authors contributed to manuscript revision, read, and approved the submitted version.

## Conflict of Interest

The authors declare that the research was conducted in the absence of any commercial or financial relationships that could be construed as a potential conflict of interest.
